# The Addition of Peripheral Blood Inflammatory Indexes to Nomogram Improves the Predictive Accuracy of Survival in Limited-Stage Small Cell Lung Cancer Patients

**DOI:** 10.3389/fonc.2021.713014

**Published:** 2021-10-08

**Authors:** Jing Qi, Jiaqi Zhang, Xingping Ge, Xin Wang, Liming Xu, Ningbo Liu, Lujun Zhao, Ping Wang

**Affiliations:** ^1^ Department of Biochemistry and Molecular Biology, Tianjin Medical University Cancer Institute and Hospital, Key Laboratory of Cancer Prevention and Therapy, National Clinical Research Center for Cancer, Tianjin’s Clinical Research Center for Cancer, Tianjin, China; ^2^ Department of Radiation Oncology, Tianjin Medical University Cancer Institute and Hospital, Key Laboratory of Cancer Prevention and Therapy, National Clinical Research Center for Cancer, Tianjin’s Clinical Research Center for Cancer, Tianjin, China; ^3^ Department of Radiation Oncology, Yantaishan Hospital, Yantai, China

**Keywords:** nomogram, inflammation, immunity, biomarker, limited-stage small cell lung cancer

## Abstract

**Background:**

Accumulated evidence for systemic inflammation response in several solid tumors prompts a possibility of prediction of patients’ prognosis in a more accessible and valuable manner. However, the prognostic value of peripheral blood inflammatory markers in limited-stage small cell lung cancer (LS-SCLC) remains unclear. Therefore, we investigated the prognostic values of pretreatment inflammatory indexes in LS-SCLC patients.

**Methods:**

We retrospectively identified 334 patients with LS-SCLC and collected their pretreatment serum levels of neutrophil, platelet, lymphocyte, leukocyte, hemoglobin, and albumin, then neutrophil-to-lymphocyte ratio (NLR), platelet-to-lymphocyte ratio (PLR), and systemic inflammation index (SII) were calculated. Patients were dichotomized as low-Risk or high-Risk group based on their corresponding cutoff values. Univariate and multivariate analyses were conducted with a Cox proportional hazards model. The least absolute shrinkage and selection operator (LASSO)-Cox regression analysis was performed to construct the inflammation-related prognostic scoring system named Risk for OS. Nomograms were established to provide prognostic information, allowing for more individualized prediction of survival.

**Results:**

Higher pretreatment platelet, lymphocyte, and albumin were indicators of favorable overall survival (OS), whereas higher NLR and SII were accompanied by inferior OS. The prognosis of patients with high Risk was significantly worse than that with low Risk in both the training group and the validation group (both p < 0.001). Comparable area under the curve (AUC) values between the training group and the validation group were observed, yielding 1-, 3-, and 5-year OS rates of 67.3% vs. 69.2%, 66.8% vs. 69.5%, and 66.7% vs. 71.4%, respectively. Multivariate analyses revealed that Risk [hazard ratio (HR) = 0.551, p < 0.001] was an independent negative prognostic indicator for OS, which was further verified in the validation set. The addition of Risk to nomogram (C-index = 0.643) harbored improved predictive accuracy for OS when compared with that of clinical factors alone (C-index = 0.606); the AUC values of 1-, 3-, and 5-year OS rates were 71.7% vs. 66.4%, 73.5% vs. 66.6%, and 71.9% vs. 65.6%, respectively.

**Conclusions:**

Pretreatment peripheral blood inflammatory indexes may be a noninvasive serum biomarker for poor prognosis in LS-SCLC. The addition of Risk to the nomogram model could serve as a more powerful, economical, and practical method to predict survival for patients with LS-SCLC.

## Introduction

Small cell lung cancer (SCLC) accounts for about 13%–15% of all newly diagnosed lung cancers globally and is characterized by rapid progression, early metastasis, and poor outcome (1). Approximately 30% of SCLC is initially categorized as limited-stage small cell lung cancer (LS-SCLC), with a median survival of 16–24 months (2). The combination of chemotherapy, thoracic radiotherapy, and prophylactic cranial irradiation (PCI), recommended by the National Comprehensive Cancer Network (NCCN) guidelines as the standard and homogeneous treatment strategy for LS-SCLC, has led to a large life span of patients. However, less attempts have been made to investigate whether clinical variables could contribute to the selection for more superior prognosis and combined modality of chemoradiotherapy of patients with LS-SCLC.

The prominent value of immune and inflammatory responses on tumor progression and patients’ prognosis is well documented in the context of several cancers (3–5). Elevated platelet counts, neutrophil-to-lymphocyte ratio (NLR), platelet-to-lymphocyte ratio (PLR), and systemic inflammation index (SII) have been found to be remarkably correlated with adverse survival of patients in cervical, colorectal, breast, and non-small cell lung cancers (6–11). However, few studies have investigated the prognostic implications of inflammatory markers among patients diagnosed with LS-SCLC (12). Recently, research has declared that higher NLR and PLR were associated with inferior prognosis and treatment efficacy in non-SCLC patients treated with immune-checkpoint inhibitors (13–15), which provided insights into whether inflammatory markers were also indicative of the selection of immunotherapy subsets in patients with SCLC, since immunotherapy has also been recommended in SCLC (16).

In this research, the least absolute shrinkage and selection operator (LASSO)-Cox regression analysis was performed to establish an inflammation-related prognostic scoring system named Risk in LS-SCLC. Additionally, combined nomogram models, including clinical variables or clinical variables plus Risk, were developed to compare the predictive accuracy of survival in patients with LS-SCLC.

## Materials and Methods

### Patient Selection

Medical records of total patients with LS-SCLC treated in Tianjin Medical University Cancer Institute and Hospital between 2008 and 2015 were retrospectively reviewed after approval by the ethics committee of our hospital, with an ethical approval number of bc2021104. The inclusion criteria were as follows: (1) patients with histopathologically confirmed SCLC; (2) patients have been reevaluated with the Veterans Administration Lung Study Group (VALG) staging standard (2017 NCCN guidelines); (3) patients who received concurrent or sequential chemoradiotherapy; and (4) patients with pretreatment computed tomography (CT) of chest and upper abdomen, positron emission tomography (PET)-CT, and laboratory tests including routine blood test and hepatic and renal function test data were all available. Patients with hematological disorders and those with active infection were excluded.

### Chemotherapy

All patients received concurrent or sequential chemoradiotherapy, and 320 patients received induction chemotherapy. The chemotherapy consisted of EP or CE regimen (EP: etoposide 100 mg, d1–5 + cisplatin 30 mg/m^2^, d1–3; CE: carboplatin 500 mg, d1+ etoposide 100 mg, d1–5). The median chemotherapeutic cycles for patients with induction chemotherapy, concurrent chemotherapy were 2 (range = 1–8) and 2 (range = 1–6), respectively. Here, 281 patients received a median of two cycles of adjuvant chemotherapy with the same regimen.

### Radiation Therapy

All patients in this study underwent radical intensity-modulated radiation therapy (IMRT). Enhanced CT scans were performed for positioning before radiotherapy, and then the radiotherapy target was delineated by Pinnacle3 8.0-m planning system. The gross tumor volume (GTV) consisted of radiographically visible lung tumors and positive lymph nodes. The clinical target volume (CTV) was derived from the GTV with a 0.5-cm margin, as well as the drainage area of each positive lymph node. The planned target volume (PTV) was defined as 0.5 cm outside the CTV and planning gross target volume (PGTV) was shaped from GTV with a margin of 0.5–1.0 cm. The radiotherapy plannings were achieved by 95% coverage on PTV of the prescription dose. The median prescription dose was 60 Gy (range = 40–66 Gy), and the dose was delivered in 20–33 fractions (median = 30). The target doses to the normal tissue constraints were <45 Gy for the spinal cord, an average of <13 Gy for total lung, and lung V20% ≤30%, lung V30% ≤20%, heart V30 ≤40%, and heart V40 ≤30%.

### Inflammatory Indices

Laboratory examinations including routine blood test and hepatic and renal function test data of patients were collected before initial treatment. The calculation formulas of NLR, PLR, and SII were as follows: NLR = neutrophil number (10^9^/L)/lymphocyte count (10^9^/L); PLR = number of platelets (10^9^/L)/number of lymphocytes (10^9^/L); SII = number of platelets (10^9^/L) × number of neutrophils (10^9^/L)/number of lymphocytes (10^9^/L). For binary group comparisons, patients in each group were dichotomized as low-Risk or high-Risk groups according to their corresponding optimal cutoff value.

### Follow-Up

Patients were followed every 3 months during the first 2 years after radiotherapy, every 6 months after 2 years of treatment, and annually thereafter. Ultrasonography, enhanced CT, magnetic resonance imaging (MRI), or PET-CT were used to evaluate the treatment efficacy during follow-up. The primary endpoint of this study was overall survival (OS), which was defined as the time from the initiation of treatment to death or last follow-up. The secondary endpoint was progression-free survival (PFS), defined as the time from treatment initiation to the first disease progression or last follow-up. Radiotherapy-related toxicities were assessed by CTCAE4.0 (Common Terminology Criteria for Adverse Events v4.0).

### Statistical Analyses

All data were analyzed using SPSS 24.0 software (IBM Corporation, Armonk, NY, USA) and R software (version 3.6.2). Chi-square test was used to compare categorical variables. Continuous variables of normal distribution and non-normal distribution were compared by independent T-test and Mann–Whitney U-test, respectively. Kaplan–Meier method was performed to delineate survival curves with Log-rank test. The optimal cutoff values of these inflammatory markers were determined using the package “maxstat” of R software based on OS. Univariate and multivariate analyses were conducted with a Cox proportional hazards model using a backward–forward stepwise method. LASSO-Cox regression analysis was performed to select the optimal prognostic factors by using the package “glmnet” of R software, and variance inflation factors (VIFs) were used to evaluate multicollinearity among variables. Heatmap was performed using the package “pheatmap” of R software.

Nomograms, including clinical variables or clinical variables plus Risk, were constructed by using the package “rms” of R software. Time-dependent receiver operating characteristic (ROC) curves and decision curve analysis (DCA) were formulated to evaluate the prognostic accuracy and clinical practicality of constructed models using package “survivalROC” and “ggDCA” of R software, respectively. Calibration index (C-index) was evaluated to assess the consistency between the predicted and observed probabilities. Delong’s test was used to compare the performance of two ROC curves. p-value ≤0.05 of two-sided test was considered indicative of significant statistical difference.

## Results

### Patient Characteristics

The distribution of clinical characteristics of 334 LS-SCLC patients is summarized in **Table 1**. In all patients, the ratio of female to male patients was 1.0:2.7; the median age was 59 years (range = 23–80 years). Two hundred sixty-eight (80.2%) patients received concurrent chemoradiotherapy after diagnosis, and 66 (19.8%) patients received sequential chemoradiotherapy. The median values of neutrophil, platelet, lymphocyte, leukocyte, hemoglobin, albumin, NLR, PLR, and SII were 4.08 × 10^9^/L (range = 0.35–12.71 × 10^9^/L), 250 × 10^9^/L (range = 95–558 × 10^9^/L), 1.75 × 10^9^/L (range = 0.43–4.84 × 10^9^/L), 6.67 × 10^9^/L (range = 1.46–15.14 × 10^9^/L), 139 × 10^9^/L (range = 84–177 g/L), 43 × 10^9^/L (range = 13.70–52.70 g/L), 2.24 (range = 0.27–17.17), 139.54 (range = 48.85–519.23), and 547.11 (range = 27.13–6,186.55), respectively.

**Table 1:** Distribution of clinical characteristics of patients with limited-stage small cell lung cancer.

Characteristics	No. of patients (%)	Characteristics	No. of patients (%)
Gender		N stage	
Male	243 (72.8)	N0	32 (9.6%)
Female	91 (27.2)	N1	34 (10.2%)
Age (years)		N2	194 (58.0%)
<65	252 (75.4)	N3	74 (22.2%)
≥65	82 (24.6)	PCI	
Smoking history		Yes	129 (38.5%)
Yes	232 (69.4)	No	205 (61.5%)
No	102 (30.6)	Induction chemotherapy	
Weight loss		Yes	320 (95.8%)
Yes	78 (23.5)	No	14 (4.2%)
No	256 (76.5)	Adjuvant chemotherapy	
KPS score		Yes	239 (71.5%)
≥80	283 (84.6)	No	95 (28.5%)
<80	51 (15.4)	Prescription dose (Gy)	
T stage		<60	100 (29.9)
T1	40 (12.0)	≥60	234(70.1)
T2	122 (36.5)	Treatment strategy	
T3	102 (30.5)	Concurrent chemoradiotherapy	268 (80.2)
T4	70 (21.0)	Sequential chemoradiotherapy	66 (19.8)

KPS, Karnofsky Performance Status; PCI, prophylactic cranial irradiation.

### Survival Outcomes

In all patients, the median follow-up length was 55.9 months, and 163 patients (60.8%) died during follow-up. The median OS and PFS were 25.7 and 12.1 months; the 1-, 3-, and 5-year OS rates were 78.4%, 40.9%, and 34.4%, respectively, and the 1-, 3-, and 5-year PFS rates were 49.1%, 28.7%, and 25.1%, respectively. The corresponding cutoff values of each inflammatory variable are shown in **Table 2**. Patients with available OS data were randomly assigned to the training set (N = 208) and the validation set (N = 91) in a ratio of 7:3. In the training set, higher pretreatment platelet (p = 0.028), lymphocyte (p = 0.012), and albumin (p = 0.004) were indicators of favorable OS, whereas higher NLR (p = 0.048) and SII (p = 0.043) were accompanied by inferior OS.

**Table 2:** Univariate Cox analyses for OS of inflammation-related factors in the training group.

Characteristics	Cutoff	Categories	RR	p-value
Neutrophil	2.07	High (≥2.07) vs. Low (<2.07)	1.801	0.108
Platelet	231	High (≥231) vs. Low (<231)	0.67	0.028
Lymphocyte	1.45	High (≥1.45) vs. Low (<1.45)	0.620	0.012
Leukocyte	6.23	High (≥6.23) vs. Low (<6.23)	0.783	0.183
Hemoglobin	130	High (≥130) vs. Low (<130)	0.767	0.156
Albumin	44.4	High (≥44.4) vs. Low (<44.4)	0.571	0.004
NLR	1.41	High (≥1.41) vs. Low (<1.41)	1.752	0.048
PLR	265.31	High (≥265.31) vs. Low (<265.31)	1.529	0.089
SII	775.58	High (≥775.58) vs. Low (<775.58)	1.454	0.043

NLR, neutrophil-to-lymphocyte ratio; OS, overall survival; PLR, platelet-to-lymphocyte ratio; SII, Systemic Inflammation Index.

### Risk Construction for Overall Survival

**Figure 1:**
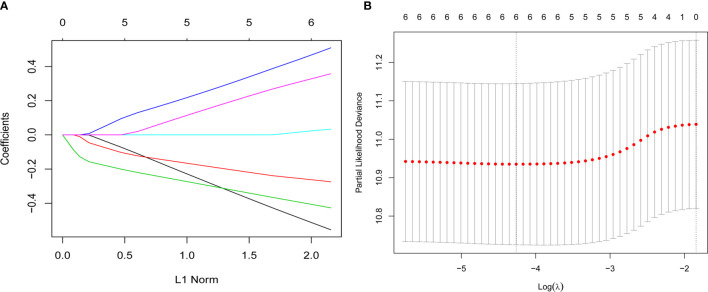
Construction of the Risk by the least absolute shrinkage and selection operator (LASSO) model in the training cohort. **(A)** The LASSO-Cox regression model was used to generate the prognostic scoring system named Risk. **(B)** Ten-fold cross-validation for tuning parameter selection in the LASSO model *via* minimum criteria and 1-SE criteria. Herein, a value λ = 0.014 with log (λ) = -4.262 was selected by minimum criteria.

**Figure 2:**
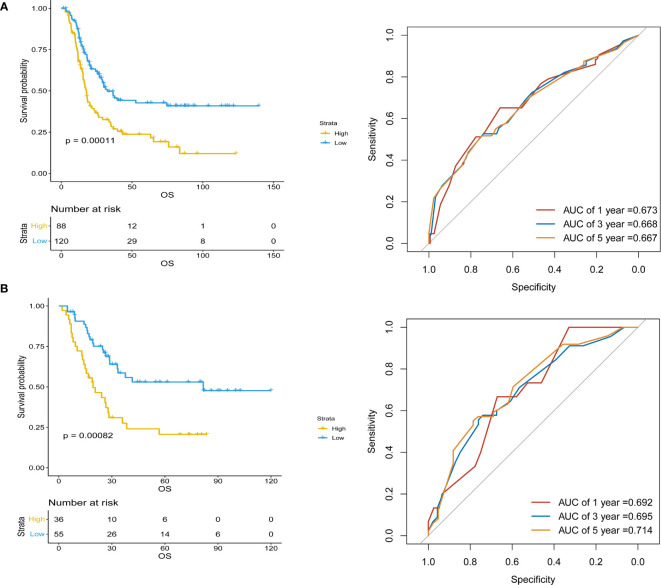
Kaplan–Meier survival analyses of Risk and Risk performance in time-dependent receiver operating characteristic (ROC) curves in **(A)** training and **(B)** validation cohorts.

**Figure 3:**
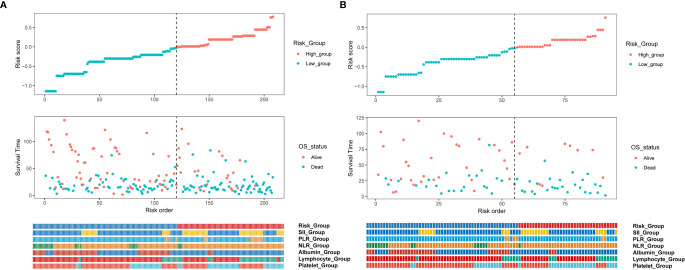
Cox regression risk score distribution, prognostic relationship, and heatmap of the dichotomous data of the inflammatory components in Risk from **(A)** training and **(B)** validation cohorts. NLR, neutrophil-to-lymphocyte ratio; PLR, platelet-to-lymphocyte ratio; SII, Systemic Inflammation Index.

Inflammatory variables with p-values <0.1 in univariate Cox analyses for OS of the training cohort were included into the LASSO-Cox regression model to formulate the prognostic scoring system (**Figure 1**). A formula named Risk was constructed as follows: Risk = -0.5745*Platelet – 0.2792*Lymphocyte – 0.4366*Albumin + 0.5280*NLR + 0.0374*PLR + 0.3708*SII. There is no collinearity among the chosen variables with the evidence of all VIF values <5. Based on “maxstat” for OS, 0 was chosen as the Risk cutoff to classify patients into low-Risk or high-Risk group. In the training set, the median OS was 36.5 and 17.7 months (p < 0.001) for low-Risk and high-Risk group, respectively, and this significant prognostic difference was also observed in the validation set (**Figure 2**). The prognostic accuracy of Risk was evaluated by the area under the curve (AUC) using time-dependent ROC analyses, yielding comparable AUC values between the training group and the validation group with 1-, 3-, and 5-year OS rates of 67.3% vs. 69.2%, 66.8% vs. 69.5%, and 66.7% vs. 71.4%, respectively, which confirmed the excellent prognostic power of Risk in another heterogeneous population. **Figure 3** shows the heatmaps of the dichotomous data of the inflammatory components in Risk from the training group and the validation group. The distribution of clinical characteristics of patients grouped by Risk in the training group and the validation group is shown in **Table 3**.

**Table 3:** Comparison of clinical characteristics of patients dichotomized by the Risk in the training and validation groups.

Characteristics	Training group (*N* = 208)				Validation group (*N* = 91)			
	*N*	Low-Risk (%)	High-Risk (%)	p-value	*N*	Low-Risk (%)	High-Risk (%)	p-value
Gender				0.65				0.948
Male	143	81 (56.6)	62 (43.4)		73	44 (60.3)	29 (39.7)	
Female	65	39 (60)	26 (40)		18	11 (61.1)	7 (38.9)	
Age (years)				0.005				0.312
<65	151	96 (63.6)	55 (36.4)		73	46 (63.0)	27 (37.0)	
≥65	57	24 (42.1)	33 (57.9)		18	9 (50.0)	9 (50.0)	
Smoking history				0.781				0.588
Yes	142	81 (57.0)	61 (43.0)		68	40 (58.8)	28 (41.2)	
No	66	39 (59.1)	27 (40.9)		23	15 (65.2)	8 (34.8)	
Weight loss				0.015				0.467
Yes	51	22 (43.1)	29 (56.9)		24	16 (66.7)	8 (33.3)	
No	157	98 (62.4)	59 (37.6)		67	39 (58.2)	28 (41.8)	
KPS score				0.208				0.19
≥80	169	94 (55.6)	75 (44.4)		72	46 (63.9)	26 (36.1)	
<80	39	26 (66.7)	13 (33.3)		9	9 (47.4)	10 (52.6)	
T stage				0.449				0.932
T1–2	108	65 (60.2)	43 (39.8)		45	27 (60.0)	18 (40.0)	
T3–4	100	55 (55.0)	45(45.0)		46	28 (60.9)	18 (39.1)	
N stage				0.892				0.072
N0–2	155	89 (57.4)	66 (42.6)		74	48 (64.9)	26 (35.1)	
N3	53	31 (58.5)	22 (41.5)		17	7 (41.2)	10 (58.8)	
PCI				0.000				0.031
Yes	132	90 (68.2)	42 (31.8)		53	37 (69.8)	16 (30.2)	
No	76	30 (39.5)	46 (60.5)		38	18 (47.4)	20 (52.6)	
Induction chemotherapy				1				0.209
Yes	200	115 (57.5)	85 (42.5)		85	53 (62.4)	32 (37.6)	
No	8	5 (62.5)	3 (37.5)		6	2 (33.3)	4 (66.7)	
Adjuvant chemotherapy				0.553				0.088
Yes	151	89 (58.9)	62 (41.1)		67	44 (65.7)	23 (34.3)	
No	57	31 (54.4)	26 (45.6)		24	11 (45.8)	13 (54.2)	
Prescription dose (Gy)				0.681				0.165
<60	70	39 (55.7)	31 (44.3)		26	18 (72.0)	7 (28.0)	
≥60	138	81 (58.7)	57 (41.3)		66	37 (56.1)	29 (43.9)	
Treatment strategy				0.727				0.97
Concurrent chemoradiotherapy	177	103 (58.2)	74 (41.8)		76	46 (60.5)	30 (39.5)	
Sequential chemoradiotherapy	31	17 (54.8)	14 (45.2)		15	9 (60)	6 (40)	

KPS, Karnofsky Performance Status; PCI, prophylactic cranial irradiation.

### Univariate and Multivariate Cox Analyses

In the training group, univariate analyses of Risk and clinical variables were performed to recognize variables associated with OS or PFS, and variables with p-values <0.1 were selected into further multivariate Cox regression analyses (**Table 4**). After adjusting clinical characteristics, Risk [hazard ratio (HR) = 0.551, p < 0.001] remained as an independent negative prognostic indicator for OS in the multivariate analyses, which was further verified in the validation set. Besides Risk, PCI (HR = 2.021, p < 0.001) and chemoradiotherapy modality (HR = 2.306, p = 0.016) were independent prognostic factors for OS. However, the significant effect of Risk on PFS was not observed in the multivariate analyses both in the training set and the validation set.

**Table 4:** Univariate and multivariate Cox analyses of baseline characteristics and Risk on survival in limited-stage small cell lung cancer patients.

Characteristics	Univariate analyses				Multivariate analyses			
	Training group		Validation group		Training group		Validation group	
	RR (95% CI)	p-value	RR (95% CI)	p-value	RR (95% CI)	p-value	RR (95% CI)	p-value
**OS**								
Gender (male vs. female)	1.203 (0.819–1.767)	0.345	1.555 (0.729–3.315)	0.253	—	—	—	—
Age (years, ≥65 vs. <65)	1.866 (1.284–2.71)	0.001	1.012 (0.518–1.976)	0.789	—	—	—	—
Smoking (yes vs. no)	1.43 (0.964–2.121)	0.076	2.027 (0.984–4.174)	0.055	—	—	2.104 (1.021–1.340)	0.044
Weight loss (yes vs. no)	0.977 (0.651–1.466)	0.909	0.711 (0.371-1.361)	0.303	—	—	—	—
KPS score (≥80)	1.042 (0.666–1.629)	0.858	1.338 (0.711–2.521)	0.367	—	—	—	—
T stage (T3–4 vs. T1–2)	1.094 (0.77–1.555)	0.615	1.499 (0.857–2.622)	0.156	—	—	—	—
N stage (N3 vs. N0–2)	0.957 (0.637–1.437)	0.832	0.743 (0.349–1.585)	0.443	—	—	—	—
PCI (yes vs. no)	0.451 (0.317–0.642)	0.000	0.544 (0.312–0.949)	0.032	2.021 (1.410–2.896)	0.000	—	—
Induction chemotherapy (yes vs. no)	1.782 (0.657–4.83)	0.256	2.313 (0.561–9.537)	0.246	—	—	—	—
Adjuvant chemotherapy (yes vs. no)	0.552 (0.375–0.812)	0.003	1.191 (0.577–2.457)	0.636	—	—	—	—
Prescription dose (Gy, ≥60 vs. <60)	0.671 (0.468–0.962)	0.030	1.075 (0.577–2.002)	0.819	—	—	—	—
Treatment strategy (concurrent vs. sequential)	1.988 (1.007–3.924)	0.048	1.164 (0.493–2.747)	0.729	2.306 (1.165–4.564)	0.016	—	—
Risk (low vs. high)	0.506 (0.356–0.720)	0.000	0.396 (0.226–0.694)	0.001	0.551 (0.384–0.790)	0.001	0.387 (0.221–0.679)	0.001
**PFS**								
Gender (male vs. female)	1.410 (0.958–2.075)	0.082	1.324 (0.679–2.583)	0.446	1.644 (1.102–2.453)	0.015	—	—
Age (years, ≥65 vs. <65)	1.354 (0.919–1.996)	0.125	0.833 (0.406–1.711)	0.619	—	—	—	—
Smoking (yes vs. no)	1.373 (0.932–2.021)	0.109	1.869 (0.974–3.586)	0.060	—	—	—	—
Weight loss (yes vs. no)	0.898 (0.598–1.347)	0.603	0.915 (0.505–1.657)	0.769	—	—	—	—
KPS score (≥80)	1.181 (0.767–1.82)	0.450	1.053 (0.557–1.991)	0.874	—	—	—	—
T stage (T3–4 vs. T1–2)	0.970 (0.685–1.374)	0.964	1.688 (0.967–2.947)	0.065	—	—	—	—
N stage (N3 vs. N0-2)	1.061 (0.707–1.591)	0.776	0.975 (0.457–2.079)	0.947	—	—	—	—
PCI (yes vs. no)	0.489 (0.343–0.697)	0.000	0.478 (0.274–0.831)	0.009	0.462 (0.321–0.665)	0.000	0.494 (0.282–0.864)	0.013
Induction chemotherapy (yes vs. no)	0.930 (0.295–2.935)	0.903	1.525 (0.210–11.066)	0.677	—	—	—	—
Adjuvant chemotherapy (yes vs. no)	0.688 (0.464–1.021)	0.063	1.048 (0.492–2.233)	0.902	—	—	—	—
Prescription dose (Gy, ≥60 vs. <60)	0.719 (0.498–1.039)	0.079	0.944 (0.515–1.727)	0.851	—	—	—	—
Treatment strategy (concurrent vs. sequential)	0.953 (0.536–1.693)	0.869	0.885 (0.350–2.242)	0.797	—	—	—	—
Risk (low vs. high)	0.568 (0.400–0.807)	0.005	0.559 (0.324–0.997)	0.037	—	—	—	—

KPS, Karnofsky Performance Status; OS, overall survival; PCI, prophylactic cranial irradiation; PFS, progression-free survival.

### Nomogram Models for Predicting Overall Survival

**Figure 4:**
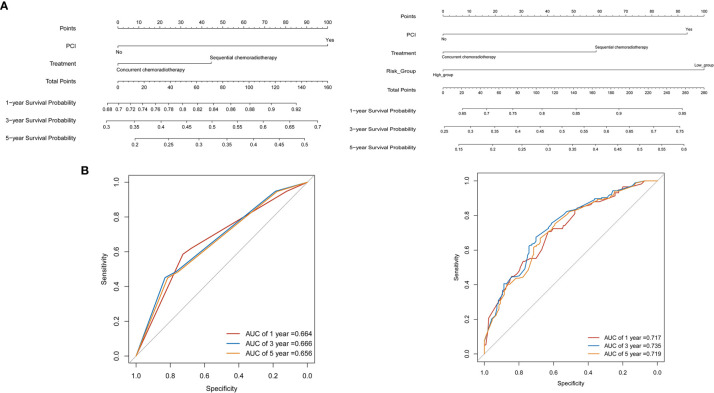
Nomograms for predicting the 1-, 3-, 5-year survival probability in limited-stage small cell lung cancer patients. **(A)** Nomograms locate each patient’s value on each variable axis, draw a line straight upward to the “Point” axis to determine the number of points received for each variable value, sum the scores achieved for each covariate, and locate the gross score on the “Total Points,” then delineate a line straight down to determine the probability of 1-, 3-, 5-year survival. **(B)** The receiver operating characteristic (ROC) curve analyses revealed the accuracy of prognosis in two nomogram models.

**Figure 5:**
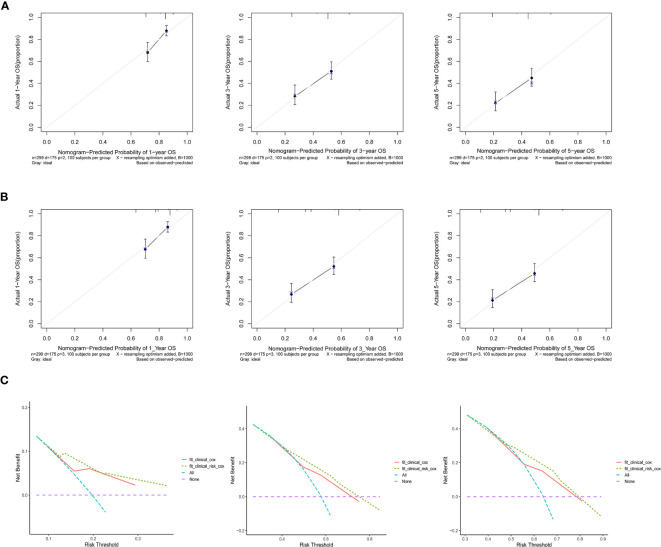
Calibration curves revealed the prediction effects of the nomogram graphs using **(A)** clinical variables and **(B)** Risk and clinical variables. **(C)** Decision curve analyses showed the net clinical benefits of the two nomograms.

Based on the multivariate regression model of the training set, nomogram models were established using clinical variables that displayed significant impact (p < 0.05) on OS with or without Risk (**Figure 4A**). The addition of Risk to nomogram (C-index = 0.643) harbored improved predictive accuracy for OS when compared with that of clinical factors alone (C-index = 0.606). The ROC curve analyses were delineated in **Figure 4B**. The AUC values of 1-, 3-, and 5-year OS rates were 66.4% vs. 71.7% (p = 0.067), 66.6% vs. 73.5% (p = 0.003), and 65.6% vs. 71.9% (p = 0.006) for the nomogram formed from clinical factors and the nomogram integrated with clinical variables and Risk, respectively. The calibration plots for the probability of OS exhibited excellent consistency between the forecast by nomogram and actual observation in both nomogram models (**Figures 5A, B** **)**. DCA curve analyses revealed that the integrated nomogram’s net clinical benefit was significantly superior to the clinical nomogram (**Figure 5C**).

## Discussion

The relationship between inflammation-based prognostic markers and tumor progression was first proposed by Rudolf Virchow in 1909 (17). Research has clarified that NLR, PLR, and SII, which are associated with inflammation and immunity, have an important influence on tumor progression, and elevated NLR and PLR have been verified to be associated with poor prognosis in a variety of cancers (9, 18, 19). In the present study, we evaluated the prognostic relevance of neutrophil counts, platelet counts, lymphocyte counts, leukocyte counts, hemoglobin, albumin, NLR, PLR, and SII in patients with LS-SCLC. We found that patients with lower pretreatment NLR and SII had significantly prolonged OS, whereas higher baseline platelet counts, lymphocyte counts, and albumin were indicators of favorable OS. Risk, formulated with the inflammation-related prognostic scoring system by LASSO-Cox model, was identified as an independent negative prognostic indicator for OS by multivariate analyses in the training set and the validation set. Meanwhile, the addition of Risk to nomogram harbored improved predictive accuracy for OS when compared with that of clinical factors alone.

Tumor-associated inflammation was prevalent in malignant tumors and significantly related to tumor progression and patients’ outcome. In recent years, several researchers have investigated the serum-derived inflammatory markers in LS-SCLC and suggested that elevated baseline NLR and PLR were indicators of inferior survival, which could serve as specific biological characteristics to improve the prediction accuracy for survival by Nomogram (12, 20, 21). However, the existence of strong collinearity among selected variables in the multivariate analyses could bring about the opposite signs of the regression coefficient by comparison with the actual situation. Furthermore, compared with a single index, a prognostic predictive model consisting of multiple dimensions may contribute to reflect the real and complicated inflammatory condition of the human body. In this study, LASSO-Cox regression model was performed using all available hematological variables to screen out preferable indicators significantly associated with survival, and the lower VIF values revealed favorable estimation results of regression coefficients simultaneously.

The important roles of inflammation and immune response have been well documented and are closely related to tumor proliferation, infiltration, tumor angiogenesis, and metastasis (22). Lymphocyte counts, platelet counts, NLR, PLR, and SII represented immune–inflammatory microenvironment in our research. Interestingly, higher albumin was also identified as an indicator of superior survival, which suggested that cancer-related malnutrition may facilitate the phenomenon of activated systemic inflammatory reactions and immunocompromised host, yielding a poor prognosis (23). In addition, high levels of neutrophils could inhibit T-cell activation and promote tumor growth by releasing a large amount of vascular endothelial growth factor and secreting reactive oxygen species (24). The ability of circulating tumor cells to interact with platelets is deemed to confer a number of advantages for protecting their survival within the circulation and promoting successful metastasis through the secretion of platelet-derived growth factors, transforming growth factor, and vascular endothelial growth factor, which could protect tumor cells from immunological assault and evasion of immune surveillance (25, 26). Thrombocytopenia and platelet function defect are correlated with reduction of metastases in preclinical experiments (27). On the contrary, lymphocytes played a crucial role in tumor defense by inducing cytotoxic cell death and inhibiting tumor cell proliferation and migration (28), thereby dictating the host immune response to malignancy (29). High NLR and PLR are strongly associated with lymphopenia, which results in a weakened host immune reaction. SII, an integrated biomarker derived from peripheral lymphocyte, neutrophil, and platelet counts, might be better able to mirror the balance of host inflammatory and immune status and has been declared to be a cogent factor of inferior prognosis in patients with liver cancer and that the high recurrence rate in patients with elevated SII may be due to increased releasing of circulating tumor cells from the primary tumor (30). Higher pretreatment NLR, PLR, and SII were proven be potential indicators of shortened survival in many cancers (31–36), which may be due to that the higher NLR, PLR, and SII might make it difficult for patients to sufficiently mobilize the influence of actionable immunostimulatory reaction on prolonged survival.

PCI and chemoradiotherapy strategy were selected as independent clinical variables to establish the nomogram prognostic prediction model based on the result of multivariate analyses in the training set. The addition of PCI exhibited a 5.4% OS benefit and was recommended as a category 1 evidence by NCCN guidelines to be delivered in patients with at least a partial response to thoracic radiation of LS-SCLC (37, 38). The combination modality of chemotherapy and thoracic radiation remains unclear due to the discrepant results in previous studies (39–41). In this study, patients who received sequential chemoradiotherapy were associated with improved OS. Interestingly, in patients with high Risk, we found that sequential chemoradiotherapy had survival superiority on concurrent chemoradiotherapy, with a median OS of 26.6 months and 16.8 months, respectively (p = 0.048). However, no significant survival benefit of sequential chemoradiotherapy was observed in patients with low Risk, yielding a median OS of 41.5 months and 36.6 months, respectively (p = 0.256). Accordingly, Risk in our study may also serve as a predictive biomarker for contributing to select the combined modality of chemoradiotherapy in patients with LS-SCLC, and patients with high Risk were recommended to receive sequential chemoradiotherapy for better prognosis.

The nomogram consists of graphic depictions of the prediction models (42). In contrast to predictive models that assign prognosis based on risk groups, nomogram provides more comprehensive information based on a combination of characteristics, allowing for a more personalized prediction of survival, which could be regarded as a new prognostic standard (43, 44). In the present study, the predictive accuracy of nomogram with the addition of Risk was significantly improved compared with that of clinical factors alone, with AUC values of 1-, 3-, and 5-year OS rates of 66.4% vs. 71.7% (p = 0.067), 66.6% vs. 73.5% (p = 0.003), and 65.6% vs. 71.9% (p = 0.006), which could serve as a valuable tool in predicting OS for LS-SCLC patients.

The current research still has several disadvantages despite the demonstration of the prognostic significance of inflammatory indexes in patients with LS-SCLC. Several potential factors, such as socioeconomic status and genomic characteristics, might have exerted inevitable influences on the results of this single-center and retrospective study. Our observations should be confirmed in multicenter prospective studies in the future.

## Conclusions

Systemic inflammation indexes may be a routinely detected, cost-effective, and easily accessible serum biomarker for unfavorable prognosis in LS-SCLC. Patients with high Risk could significantly benefit from sequential chemoradiotherapy, and the addition of Risk to nomogram model could serve as a more powerful, economical, and practical method to predict survival in patients with LS-SCLC.

## Data Availability Statement

All data generated or analyzed during this study are included in this published article, and the primary datasets are available on request to the corresponding authors.

## Ethics Statement

The studies involving human participants were reviewed and approved by Tianjin Medical University Cancer Institute and Hospital. Written informed consent for participation was not required for this study in accordance with the national legislation and the institutional requirements.

## Author Contributions

JQ, JZ, and XG have contributed equally to this work. JQ JZ, LZ, and PW designed of the study. JQ, JZ, XG, XW, LX, and NL participated in data collection or data analyses and interpretation. JQ, JZ, XG, NL, LZ, and PW wrote this article or made critical revisions to pivotal intellectual content. All authors contributed to the article and approved the submitted version.

## Conflict of Interest

The authors declare that the research was conducted in the absence of any commercial or financial relationships that could be construed as a potential conflict of interest.

## Publisher’s Note

All claims expressed in this article are solely those of the authors and do not necessarily represent those of their affiliated organizations, or those of the publisher, the editors and the reviewers. Any product that may be evaluated in this article, or claim that may be made by its manufacturer, is not guaranteed or endorsed by the publisher.
